# Visual sense of number *vs*. sense of magnitude in humans and machines

**DOI:** 10.1038/s41598-020-66838-5

**Published:** 2020-06-22

**Authors:** Alberto Testolin, Serena Dolfi, Mathijs Rochus, Marco Zorzi

**Affiliations:** 1 Department of General Psychology and Padova Neuroscience Center, University of Padova, 35131 Padova, Italy; 2 Department of Information Engineering, University of Padova, 35131 Padova, Italy; 3 Department of Experimental Psychology, Ghent University, 9000 Ghent, Belgium; 4 IRCCS San Camillo Hospital, 30126 Venice-Lido, Italy

**Keywords:** Perception, Network models, Human behaviour, Computational science

## Abstract

Numerosity perception is thought to be foundational to mathematical learning, but its computational bases are strongly debated. Some investigators argue that humans are endowed with a specialized system supporting numerical representations; others argue that visual numerosity is estimated using continuous magnitudes, such as density or area, which usually co-vary with number. Here we reconcile these contrasting perspectives by testing deep neural networks on the same numerosity comparison task that was administered to human participants, using a stimulus space that allows the precise measurement of the contribution of non-numerical features. Our model accurately simulates the psychophysics of numerosity perception and the associated developmental changes: discrimination is driven by numerosity, but non-numerical features also have a significant impact, especially early during development. Representational similarity analysis further highlights that both numerosity and continuous magnitudes are spontaneously encoded in deep networks even when no task has to be carried out, suggesting that numerosity is a major, salient property of our visual environment.

## Introduction

It is widely believed that the cognitive foundations of numerical competence rest on basic numerical intuitions, such as the ability to discriminate sets with different numerosity or to rapidly estimate the amount of items in a display^1–3^. Discrimination between two numerosities is modulated by their numerical ratio, and the individual psychometric function provides an index of “number acuity” representing the internal Weber fraction, *w*^4^. Numerosity perception is shared with many animal species^5–7^ and in the primate brain it is supported by an occipito-parietal network^4,8^. Even human newborns and infants appear sensitive to numerosity^9,10^, although the improvement of number acuity throughout childhood suggests that learning and sensory experience play an important role in refining our numerical representations^11^. Moreover, individual differences in number acuity have been related to mathematical learning performance both in typical and atypical development^12–14^.

Nevertheless, the nature of the mechanisms underlying numerosity perception is still hotly debated. According to the “number sense” hypothesis, visual numerosity is a primary perceptual attribute^15^, spontaneously extracted^16,17^ by a system yielding an approximate representation of numerical quantity. However, it has been repeatedly pointed out that numerosity judgments can be modulated by non-numerical perceptual cues that usually co-vary with number, such as cumulative surface area^18^, total item perimeter^19^ and convex hull^20^, over which it is impossible to exert full experimental control and which can hinder numerosity discrimination when carrying incongruent information^21,22^. These findings have led to the proposal that numerosity is indirectly estimated from non-numerical visual features, thereby calling into question the existence of a dedicated system for numerosity perception^23^. Moreover, the influence of non-numerical cues is stronger in young children^24^ and in children with mathematical learning deficits^25^. In a classic “number sense” view, the developmental improvement of numerosity estimation entails progressive sharpening of the internal representation (i.e., increasing representational precision), but an alternative hypothesis is that it simply reflects the increasing ability to focus on the relevant dimension and filter out (or inhibit) irrelevant non-numerical features^22,26^.

Here we shed light on the theoretical debate regarding the mechanism supporting numerosity perception – and how it is shaped by learning and visual experience – by means of computational modeling based on deep neural networks. Besides driving the contemporary artificial intelligence revolution^27^, deep networks are being increasingly proposed as models of neural information processing in the brain^28,29^. In contrast to the mainstream *supervised* deep learning approach, which has been criticized for its limited biological and psychological plausibility^30,31^, our computational model is based on “deep belief networks”, which implement *unsupervised* learning through Hebbian-like principles^32^. In line with modern theories of cortical functioning, learning in these networks can be interpreted as the process of fitting a probabilistic, hierarchical generative model to the sensory data^33,34^. This corresponds to a form of learning by observation, where connection weights are changed according to the statistical regularities in the sensory input; it also dispenses with the implausible assumption that learning requires labeled examples, since the only objective is to discover efficient internal representations of the environment^30^. Notably, it has been shown that unsupervised deep learning can give rise to extremely high-level, abstract representations (for example, face detectors^35^ and letter features^36^).

The importance of unsupervised representation learning is particularly evident in the context of numerosity perception, which develops in infants and naive animals without explicit feedback^37,38^. Previous work has shown that deep belief networks can indeed simulate basic numerical abilities^39^. However, in the original model only cumulative area was taken into account as possible confound, and subsequent simulations presented anectodical evidence that stronger congruency manipulations can affect model’s responses^40^. Here we push this modeling approach one step further. We adopted a recently proposed “stimulus space”^41^ that allows a systematic, multi-dimensional manipulation of the non-numerical features to create images of visual sets that were used to train and test a large number (N = 144) of deep networks (which varied in number of neurons and initial weight values to ensure robustness and reproducibility of results). Crucially, for the very first time we tested human observers and deep networks on a numerosity comparison task using exactly the same visual stimuli, thus allowing for accurate simulations of psychophysical data and fine-grained assessment of the contribution of non-numerical visual features, using the methods developed in^41^. We also compared deep networks at different time points during unsupervised learning to assess how visual experience shapes the interplay between numerosity and non-numerical visual features, validating our simulations against existing developmental data collected on children using the same stimulus space^24^. Finally, we used representational similarity analysis^42^ to investigate the encoding of numerosity and continuous visual features in the deep networks’ internal representations, thereby shedding light on whether spontaneous sensitivity to number emerges (from unsupervised learning) in the absence of any explicit task.

## Results

**Figure 1:**
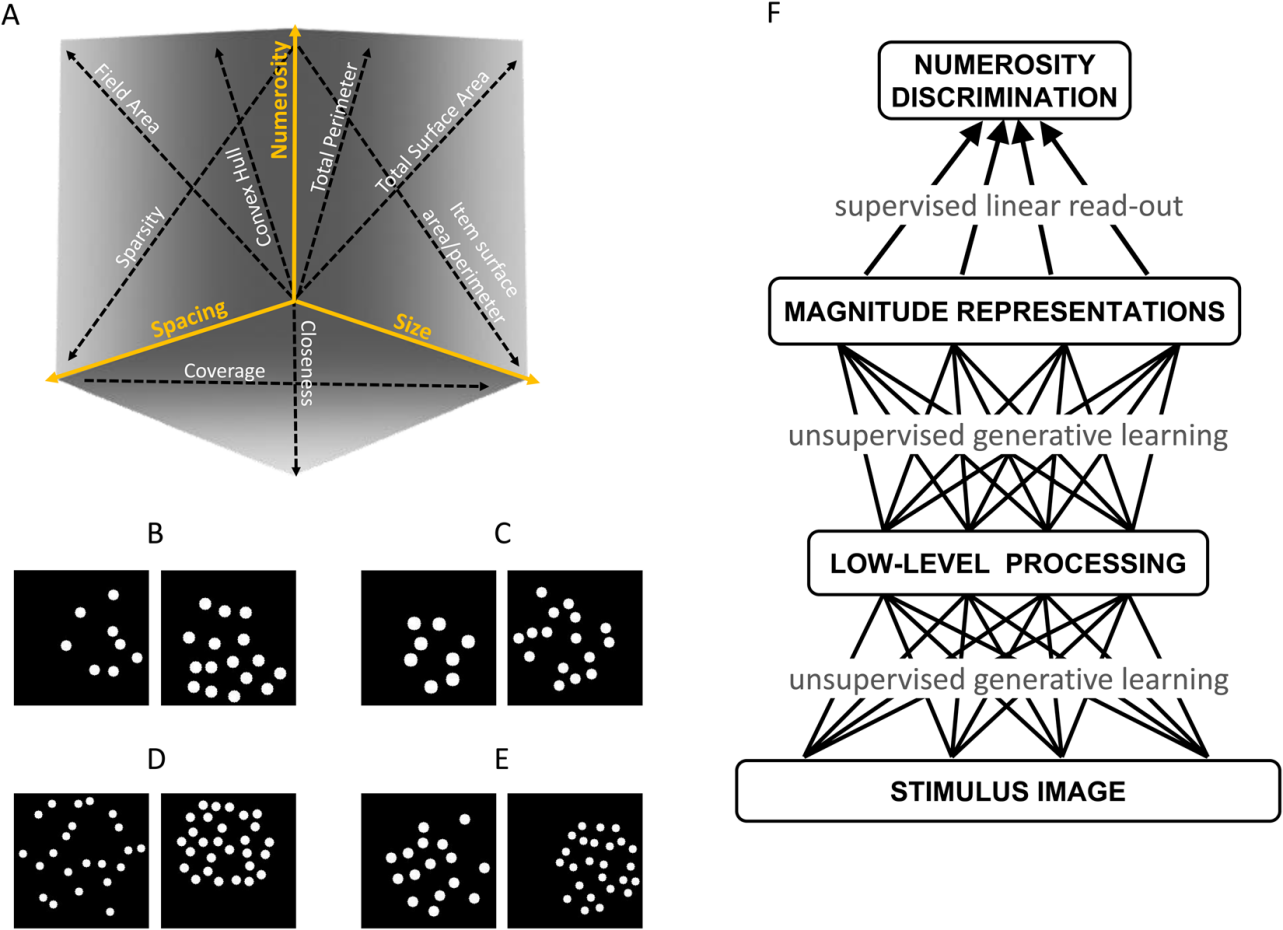
Stimulus space and model architecture. (**A**) The 3D stimulus space defined by the Numerosity, Size and Spacing orthogonal dimensions (adapted from^41^). Non-numerical features are represented as arrows to indicate the direction in which they increase, and each stimulus image can be represented as a point in this space. Example of stimuli pairs are shown below, where Numerosity can be fully congruent for Size and Spacing (**B**), congruent for Spacing but not for Size (**C**), congruent for Size but not for Spacing (**D**), or fully incongruent for Size and Spacing (**E**). The model architecture is depicted in panel (**F**). At the initial stage, unsupervised deep learning adapts the connection weights of the first two layers (undirected edges) by capturing the statistical distribution of active pixels in the images. During task learning, a supervised linear classifier adapts the connection weights of the final layer (directed edges) in order to minimize discrimination error.

Image stimuli containing clouds of dots were sampled from a multidimensional space defined by three orthogonal dimensions, representing the degrees of freedom used to generate all possible combinations of numerosity and non-numerical features in a visual display^41^. *Numerosity* corresponds to the discrete number of dots in the image, *Spacing* jointly encodes for variations in field area and density of the dots, while *Size* jointly encodes for variations in dot surface area and total surface area (see Fig. 1A–E for graphical representation and sample stimuli, and Supplementary Information for details). Humans and machines were probed using a standard numerosity comparison task that required indicating which of two images had more dots. Behavioral choices were modeled using a generalized linear model (GLM) with regressors for the log of the ratio of each orthogonal dimension (see Methods). Besides offering a better estimate of number acuity compared to traditional measures^41^, this method returns coefficients describing the contribution of each non-numerical feature in behavioral performance. For example, a large Numerosity coefficient would reflect the ability to discriminate difficult numerical ratios, while large coefficients for Size and Spacing would highlight strong biases on the participant’s choices due to changes in non-numerical features.

### Human behavioral performance

**Figure 2:**
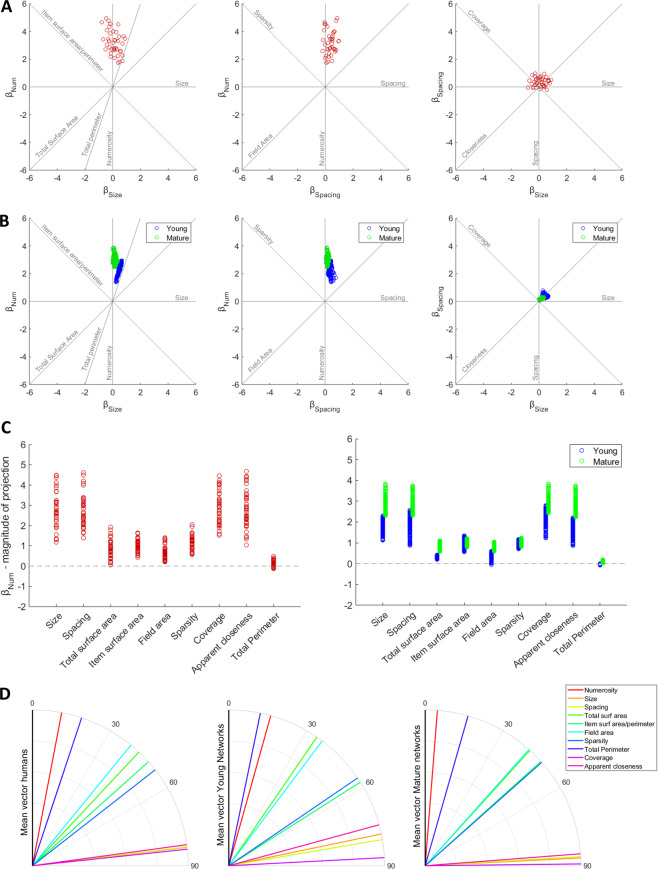
Psychophysics of numerosity comparison in humans and deep networks. Scatter plots of Numerosity, Size and Spacing coefficients for humans (**A**) and all deep networks (**B**), also showing the axes of individual features as done in^41^. (**C**) Differences between the projection on the Numerosity dimension and the projections on all individual non-numerical features for humans (left) and deep networks (right). Positive values indicate that number was a better predictor of behavior than the specific feature. Negative values would indicate that the considered feature had greater impact on discrimination choice. (**D**) Angles between the discrimination vector and all non-numerical features (the discrimination vector is on the *y* axis) for humans (left) and deep networks at two different developmental stages: Young (middle) and Mature (right).

Discrimination accuracy was well above chance (mean 83%, range: 69–91%). The GLM fit at individual level was significant (mean adjusted *R*^2^ = 0.55, mean chi-square value = 191.14, all *p* < 0.001). Coefficient fits for each orthogonal dimension were significantly different from zero for *β*_*Num*_ (*t*(39) = 23.54, *p* < 0.001) and *β*_*Spacing*_ (*t*(39) = 7.21, *p* < 0.001), but not for *β*_*Size*_ (*t*(39) = 1.37, *p* = 0.18). Coefficient estimates are shown in the scatter plots of Fig. 2A, along with the axes representing individual features. Numerosity was by far the dominant dimension in shaping participants’ choices. Nevertheless, most of the participants were also influenced by Size, Spacing, or both; only 5 participants out of 40 showed an unbiased performance (all *ts* < 1.35, *p* > 0.10 for *β*_*Size*_, and all *ts* < 1.45, *p* > 0.15 for *β*_*Spacing*_). Model fits for two representative participants are shown in Fig. S1, with black curves representing the model fit on the full dataset and colored lines representing model predictions on two subsets of congruent and incongruent trials with extreme values of Size and Spacing ratios. The offsets of the colored lines from the black lines highlight that these participants were influenced by Size (panel A) or both Size and Spacing (panel B), resulting in better discrimination for congruent trials and a decrease in performance for incongruent trials. These psychometric curves also highlight that, as expected, accuracy was much lower for hard trials (i.e., with ratio closer to 1) compared to easy trials.

In the 3-dimensional space, the coefficients estimated for the orthogonal dimensions define a discrimination vector whose projection on each individual axis indicates the strength of the influence of the corresponding feature (left panel in Fig. 2C). Paired t-tests revealed that the projection on the Numerosity dimension was significantly larger than the projection on all other individual features (all *t*(39) > 5.45, all *p* < 0.001), replicating the findings of DeWind et al. (2015). The overall pattern was confirmed by angle analysis (left panel in Fig. 2D), in which Numerosity resulted the dimension closest to the discrimination vector (10.79 deg) followed by Total Perimeter (18.39 deg); the angle between these two axes was significantly different (*Z* = 4.14, *p* < 0.001).

### Deep networks behavioral performance

The same numerosity comparison task was simulated with deep neural networks. Deep networks (N = 144) with varying architectures and initial weights (see Methods and SI) were first trained in a completely unsupervised way, using as input individual images sampled from the stimulus space^1^^1^. The objective of unsupervised deep learning was to build a generative model of the data, that is, to maximize the likelihood of reproducing samples from the input distribution. This corresponds to a form of learning by observation, where connection weights are changed according to the statistical regularities in the sensory input; no information about item numerosity was provided at this stage.

As a second step, a supervised linear network was stacked on top of the deep network: in analogy with training procedures used in animal studies, this read-out layer allowed to carry out an explicit discrimination task even in the absence of explicit (verbal) instructions. The classifier was fed with the deep network’s internal representations of two image pairs, with the objective of choosing which image had the larger numerosity (see Fig. 1F for a graphical representation of the model architecture). Testing was carried out on a separate set of images that were never seen during training and it took place at two different developmental points of the unsupervised learning phase. In the “Young” condition the network was trained for only one pass (epoch) through the entire image dataset, whereas in the “Mature” condition training was prolonged for 200 epochs: one epoch simply marks the completion of the first cycle through the training images, whereas 200 epochs represent a stage where learning has clearly converged (as indexed by asymptotic behavior of the training loss function)^2^^2^.

**Figure 3:**
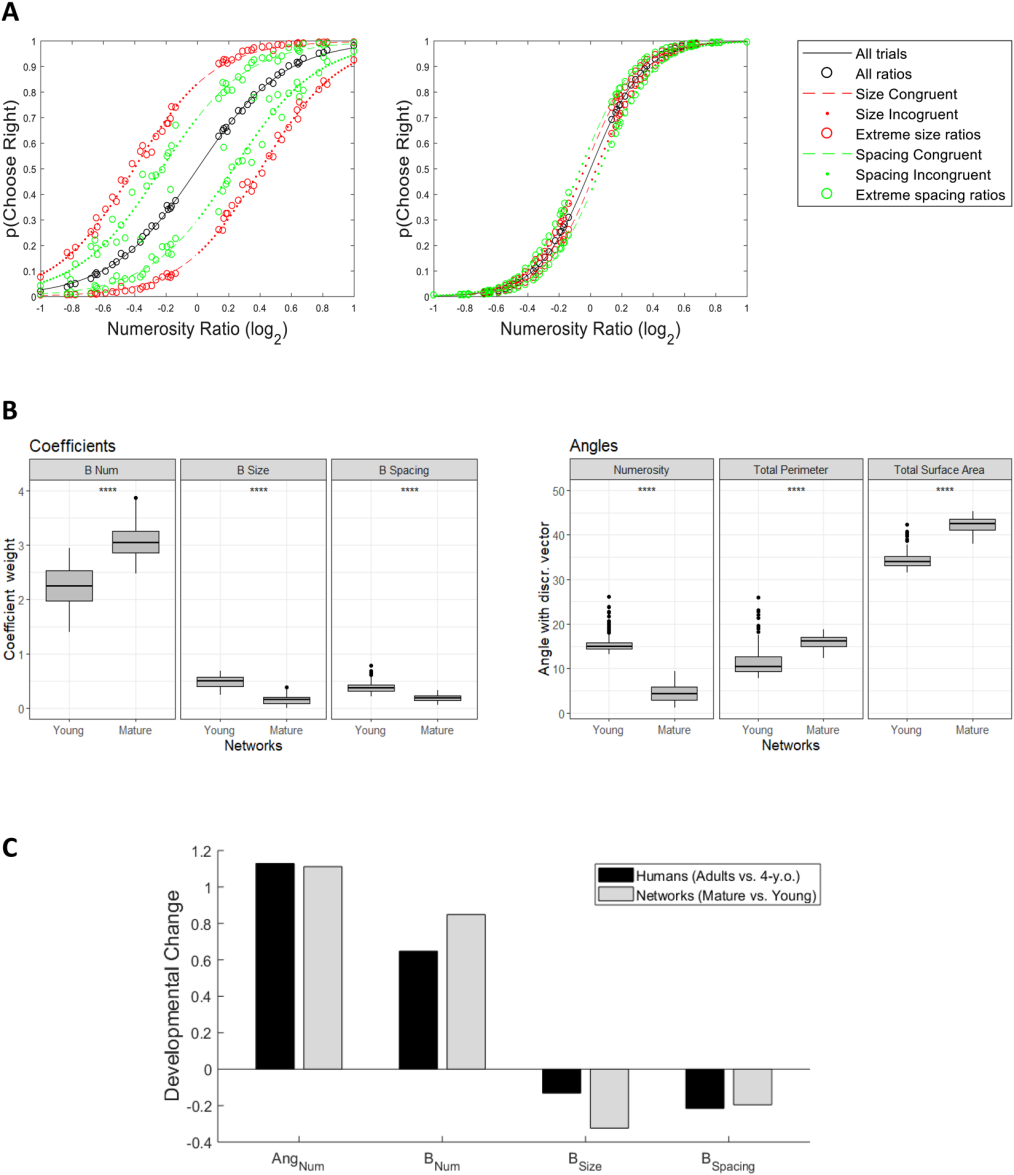
Maturation of number acuity in deep networks. (**A**) GLM fit for one Young (left panel) and one Mature (right panel) network, visualized as in^41^. Black lines indicate the model fit for all data (black circles). Red color shows data and model fits for the trials with extreme Size ratio, while green color shows data and model fit for trials with great Spacing ratio. Dashed lines indicate that Size or Spacing were congruent with numerosity, while dotted lines indicate incongruent trials. (**B**) Left panel: Differences in Numerosity, Size and Spacing coefficients, measured separately for the Young and Mature networks. Right panel: Differences in angles between the discrimination vector and the most relevant non-numerical features, measured separately for the Young and Mature networks. (**C**) Comparison between angle and coefficients changes in humans (data replotted from^24^) and deep networks. Note that angle differences for both humans and networks have been scaled by a factor of 10 for visualization purposes.

As shown in Fig. 2B, the GLM coefficients for the 144 individual deep networks were aligned with those of human observers, highlighting the primary contribution of Numerosity but also the impact of Size and Spacing in biasing the numerosity judgments. All coefficient fits were significantly different from zero both for Young networks (*β*_*Num*_ all *t* > 66, *p* < 0.001; *β*_*Spacing*_ all *t* > 17.9, *p* < 0.001; *β*_*Size*_ all *t* > 32, *p* < 0.001) and Mature networks (*β*_*Num*_ all *t* > 66, *p* < 0.001; *β*_*Spacing*_ all *t* > 6.9, *p* < 0.001; *β*_*Size*_ all *t* > 2.6, *p* < 0.001). It is important to emphasize that variability across networks was limited, thereby showing that the modeling results are robust to changes in architecture and initial state. GLM fits for representative Young and Mature networks are shown in Fig. 3A; as for the human participants reported in Fig. S1, the offsets of the colored lines from the black lines highlight that model choices were influenced by Size and Spacing, resulting in better discrimination for congruent trials and worse discrimination for incongruent trials, especially for Young networks. Note also how the psychometric curve is much steeper for the Mature network, reflecting the increase in number acuity.

As shown in Fig. 2C (right panel), for Young networks the vector projection on the Numerosity dimension was larger than the projections on the other individual features (all *Z* > 10.40, all *p* < 0.001) except for Total Perimeter, whose projection resulted higher than *β*_*Num*_ (*Z* = −10.41*, p* < 0.001). For the Mature networks, the vector projection on Numerosity was larger than all other projections (all *Z* > 10.41, all *p* < 0.001). This pattern was confirmed by angle analysis (Fig. 2D): in the Mature networks Numerosity was the dimension closest to the discrimination vector (4.49 deg) followed by Total Perimeter (15.96 deg), and the angle between these two dimensions was significantly different (*Z* = 10.41, *p* < 0.001). However, in the Young networks the closest dimension was Total Perimeter (11.69 deg), followed by Numerosity (15.61 deg), and the angle between these two dimensions was significantly different (*Z* = −10.41, *p* < 0.001).

Unsupervised learning thus seems to have strengthened the encoding of numerical information in the deep networks, supporting higher accuracy in the numerosity comparison task: Although explicit numerosity judgments in the model are affected both by unsupervised representation learning and supervised task learning, the supervised layer was exactly the same for Young and Mature models, implying that changes in coefficients derive from a refinement of the internal representations. As shown in Fig. 3B (left panel), *β*_*Num*_ was much higher for the Mature network compared to the Young network (*U* = 713, *p* < 0.001), while the influence of both Size (*U* = 20535, *p* < 0.001) and Spacing (*U* = 20393, *p* < 0.001) significantly decreased. At the level of individual features (right panel in Fig. 3B) we also observed a large difference in angles with the discrimination vector: the angle with Numerosity significantly decreased in the Mature network (*U* = 20736, *p* < 0.001), while there was a significant increase of the angles with Total Perimeter (*U* = 2623, *p* < 0.001) and Total Surface Area (*U* = 142, *p* < 0.001). These results are well aligned with recent human developmental data collected using the same stimulus space^24^: the comparison between 4-year-old children (the youngest group tested) and adults reveals a marked increase in the Numerosity coefficient and a moderate decrease of Size and Spacing coefficients, as well as a significant reduction in angle with Numerosity (see Fig. 3C). This developmental change is very similar to that observed in the deep networks between Young and Mature states. Overall, these findings suggest that numerosity is the primary feature driving numerical comparison both in children and adults, but with a different non-numerical bias that depends on the amount of sensory experience (which affects the quality of the learned generative model).

Nevertheless, it is well-known that numerosity discrimination performance can also be modulated by feedback^43^ and by the history of preceding trials^44^. In particular, numerosity discrimination improves when starting with easier trials and gradually progressing to harder ones, compared with the reverse (an effect known as perceptual hysteresis^44^; in the machine learning literature, progression from easy to hard training samples is instead known as curriculum learning^45,46^). We investigated whether the hysteresis effect can be linked to classifier training in our model, that is, to the read-out of numerical information during task execution. We implemented an iterative version of the classifier and manipulated the training sequence to reflect an increase or decrease of task difficulty during learning (see SI). This manipulation yielded a small but reliable effect in the Mature network, which achieved higher number acuity (lower Weber fraction) when classifier training started from the easiest trials (see Fig. S2 and Supplementary Table S2). However, the effect was not observed for the Young network, suggesting that further modeling work is required to account for the hysteresis effect observed in children and infants^44,47^.

### Deep networks internal encoding

We systematically analyzed the deep networks’ internal representations to disentangle the contribution of unsupervised deep learning in numerosity perception from the (supervised) task-driven decoding of information supporting numerosity judgments. To this aim we investigated how numerosity and non-numerical features were spontaneously encoded in the activation patterns of hidden neurons in response to individual images, in the absence of an explicit task that requires focusing on numerical information as a salient dimension to guide overt behavior (i.e., without considering the discrimination layer).

**Figure 4:**
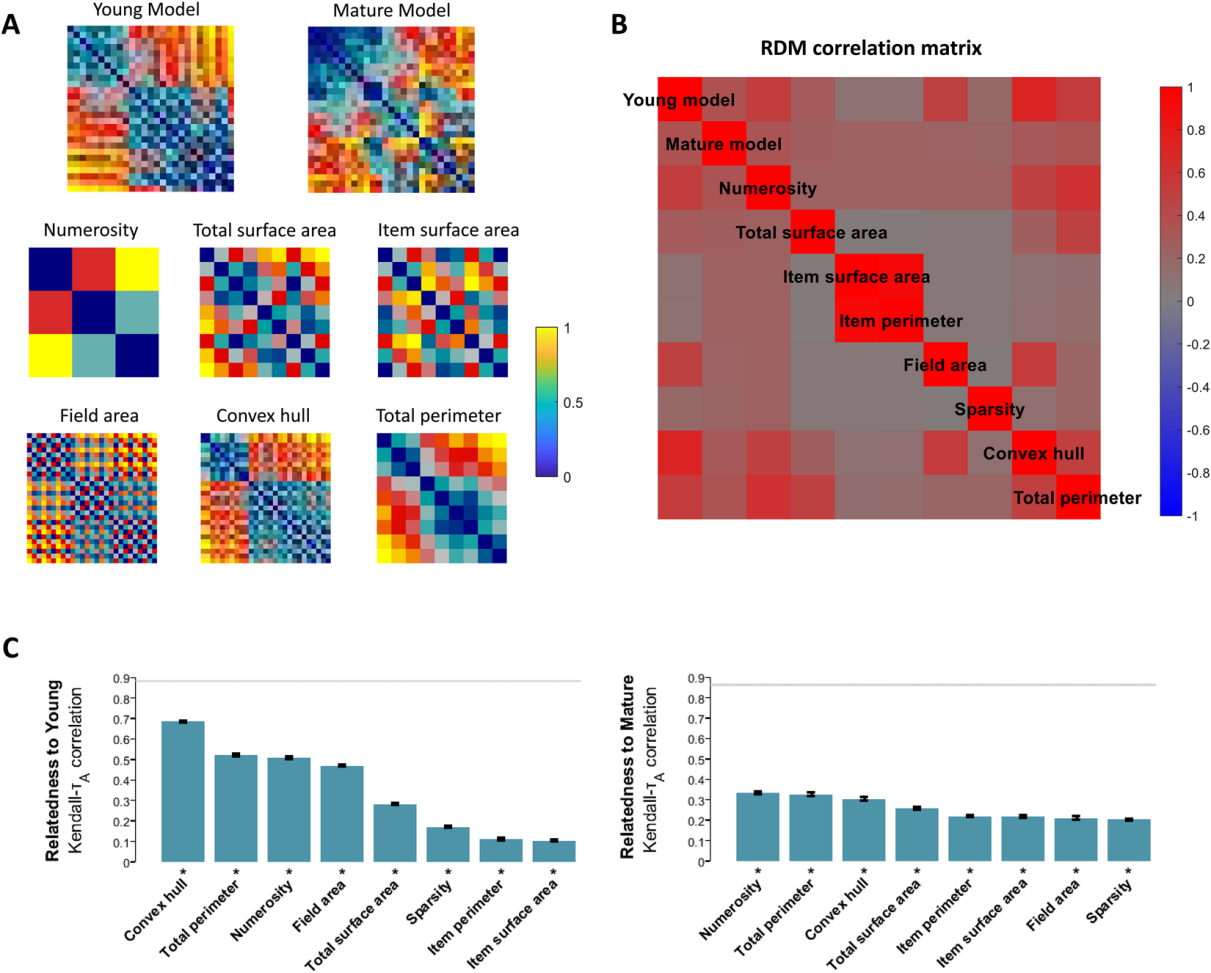
Representational similarity analysis. (**A**) Representational dissimilarity matrices for the best deep network architecture (distance measure: 1 – Pearson correlation) and the most relevant categorical models (distance measure: log distance between stimulus features). Each RDM was separately rank transformed and scaled into [0,1]. (**B**) Second-order correlation matrix showing the pairwise correlations between RDMs. (**C**) Relatedness between the model’s RDM and the categorical RDMs, measured as the Kendall rank correlation between dissimilarity matrices. Asterisks indicate significance in a one-sided signed rank test, thresholded at FDR < 0.01. Error bars indicate the standard error of the correlation estimate. Grey horizontal lines represent noise ceiling (i.e., the highest correlation that could be achieved considering the data variability).

We performed a Representational Similarity Analysis (RSA)^42^ to assess which features were encoded in the network’s internal representations. As a first step, Representational Dissimilarity Matrices (RDMs) of all deep networks were fitted with a GLM, using as predictors the three orthogonal features defining the stimulus space. In line with the task-driven simulations, the coefficient for Numerosity was far greater than those for Size [Young: *t*(11) = 117.87; Mature: *t*(11) = 10.84, *ps* < 0.001] and Spacing [Young: *t*(11) = 59.33; Mature: *t*(11) = 11.98, *ps* < 0.001]. This analysis highlights an overall decrease for the Numerosity coefficient over the course of unsupervised learning, which might be due to a more explicit encoding of other co-varying magnitudes (e.g., Total Perimeter) in Young networks. We thus compared the RDM obtained from the best performing deep network architecture with conceptual RDMs reflecting specific categorical models (Fig. 4A): The correlations between simulated RDMs and individual categorical models can be visualized as a second-order correlation matrix (Fig. 4B). The Young network exhibited a stronger correlation (Kendall Tau alpha, see Fig. 4C) with the RDM produced using Convex Hull (τ_A_ = 0.69), followed by Total Perimeter (τ_A_ = 0.52), Numerosity (τ_A_ = 0.51) and Field Area (τ_A_ = 0.47); correlation with all other categorical models was smaller, but significant in a one-sided signed rank test, thresholded at FDR < 0.01. The Mature network, instead, had stronger correlations with the RDMs for Numerosity (τ_A_ = 0.33), Total Perimeter (τ_A_ = 0.33) and Convex Hull (τ_A_ = 0.30), and significant correlations also with all the other categorical models (FDR < 0.01). Pairwise comparisons are shown in Fig. S3, highlighting the primary role of Convex Hull during early developmental stages, but an increase in the contribution of Numerosity later in development. The increase in correlation with all categorical models suggests that the representational space become more disentangled, thus allowing for a better factorization of all the latent features of the stimulus space. Control simulations (see Supplementary Table S1) indeed showed that linear decoding of Size also improved as a result of unsupervised learning; decoding of Spacing was already accurate at the Young stage.

**Figure 5:**
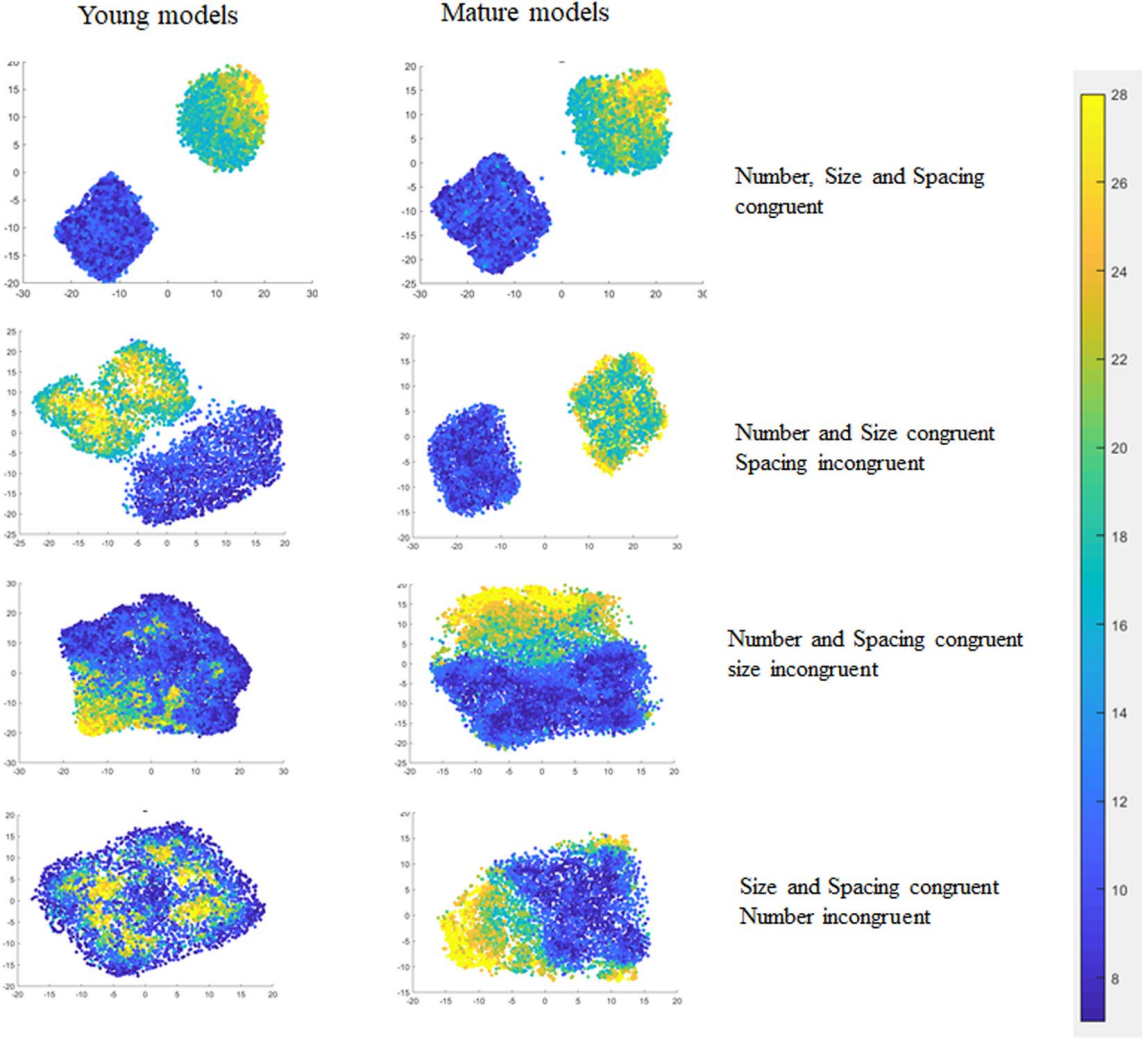
Manifold projection using t-SNE. Stimuli with a small or large numerosity (respectively in the ranges 7:12 and 16:28) were first selected from the complete image data set. In the top panels, Numerosity, Size and Spacing are all congruent, which means that images with a small number of dots also have low Spacing and Size values. In the second-row panels, Numerosity and Size are congruent, but Spacing is not. In the third-row panels, Numerosity and Spacing are congruent, but Size is not. In the bottom panels, both Spacing and Size are incongruent with Numerosity. Results show that in the Mature model the representations are mostly clustered, with a distance gradient often proportional to number. In the Young model, when number is incongruent with Size the clustering almost disappears, especially when also Spacing is incongruent.

Overall, RSA showed that numerosity information was spontaneously encoded as a salient dimension in the representational geometry of the deep network, even when no explicit numerosity judgements had to be carried out. A compatible result was obtained using t-SNE, which is a technique commonly used for visualizing the high-dimensional representational space of deep networks (see Fig. 5 and SI). When t-SNE was performed on the internal representations of images where Numerosity, Size and Spacing were all congruent, the algorithm was able to project patterns corresponding to small and large magnitudes into clearly separated clusters. When either Size or Spacing information was incongruent with number, the separation was still possible, but more evident in Mature networks. When both Size and Spacing were incongruent with number, the internal representations of Young networks did not support the formation of separate clusters.

## Discussion

The computational investigations presented in this article reconcile two contrasting perspectives about the nature of our visual number sense. On the one hand, numerosity turned out to be the primary driver of both humans’ and deep networks’ responses in a numerosity comparison task, even when non-numerical visual cues were included as predictors of behavioral choices. Numerosity was also a critical factor in shaping the internal representations emerging from unsupervised deep learning, showing that number was spontaneously encoded in the model even when no number-related decision had to be carried out (and indeed, in the absence of any task). These results support the characterization of numerosity as a primary perceptual attribute^17^ and are aligned with the hypothesis that animals are endowed with a number sense^1,3^. On the other hand, continuous visual features had a significant impact both on humans’ and deep networks’ responses, thus confirming that numerosity estimation is modulated by non-numerical magnitudes that usually co-vary with number^23,48^. This was particularly the case for Total Perimeter, which was the most influential non-numerical feature modulating human judgements and models’ responses, especially at the initial learning stage. Although this effect might be partially due to the fact that the axis for Total Perimeter is particularly close (and thus correlated) to the axis for Numerosity in the current stimulus space, this finding supports the hypothesis that ﻿the ability to perceive and evaluate sizes ﻿might play an important role in the development of numerical processing^49^. Moreover, most of the continuous magnitudes were also spontaneously encoded in the representational space of the model, suggesting that non-numerical features are equally important for capturing the latent factors of variation underlying the sensory data.

According to our model, the influence of non-numerical features might be seen as the concurrent processing of other dimensions carrying magnitude information, but without necessarily implying that numerosity is constructed out of those dimensions. In our stimulus space Numerosity, Size and Spacing varied within the same range, in order to make sure that one dimension was not statistically more salient (i.e., of higher variance) than the others. However, an interesting research direction could be to investigate how the representational space might change under different distributional properties, for example by creating stimuli that match the statistical distribution of visual features in natural environments^50^.

Our simulations show that numerosity processing can be carried out using generic low-level computations, such as those emerging in multi-layered neural networks that learn a hierarchical generative model of the sensory data. Previous modeling work has shown that high-frequency spatial filtering of the image is the building block for computing numerical information at higher levels in the neural processing hierarchy^39,40^. This is consistent with a psychophysical modeling approach based on spatial filtering^51^ and with recent electrophysiological evidence suggesting that numerosity-sensitive responses are present at early stages of visual processing^52,53^. Numerical information in the deep neural network is best conceived as a population code over the hidden neurons^39,40^, as also shown here by the representational similarity analysis. It should be emphasized that these results do not necessarily imply the existence of a system that is fully dedicated to numerosity: rather, our analyses show that numerosity can emerge as a partially disentangled factor in the latent representational space of deep networks, which is nevertheless also modulated by non-numerical magnitudes. Although sensitivity to numerosity has been found at the level of individual neurons in deep networks^39,40,54^, with tuning functions resembling those of real neurons in the dorsal visual stream of non-human primates^55^, the modulatory effect of non-numerical features in the response profiles of single-neurons awaits systematic examination both in neurophysiological studies and deep learning simulations. Moreover, one important question for future computational investigations is whether learning to count and the interactions with number symbols^56^ could promote the emergence of fully disentangled numerical representations.

It should be noted that, in accordance with previous experimental paradigms, in our setup visual exploration (i.e., eye movements) was prevented by the short display duration, which is aligned with our modeling approach based on pure parallel processing. Nevertheless, a recent study showed that approximate estimation of large numerosities can benefit from the deployment of serial accumulation mechanisms^57^, which could in principle be simulated by implementing more sophisticated, sequential network architectures^58^. However, it should also be noted that converging evidence points to the presence of separate mechanisms for perception of numerosity and texture/density when displays might contain a large number of items^59,60^.

The comparison between Young and Mature deep networks showed an overall improvement in number acuity, in line with developmental studies. This improvement reflected the increased weighting of numerical information and the concurrent down-weighting of the non-numerical dimensions^24^. Such developmental change might be interpreted in terms of an improved ability to focus on numerosity and to filter out task-irrelevant features, so that the discrimination boundary gets progressively aligned to the task-relevant dimension^26^. However, in our simulations the discrimination layer received identical training when applied to both Young and Mature networks, thereby suggesting that the improvement stems from a refinement of the internal representations following unsupervised learning. In other words, a sharper internal encoding allows a better disentanglement of numerosity from other dimensions defining the statistical structure of the visual environment. The sharpening hypothesis is also supported by our representational similarity analysis, which shows that numerosity is indeed spontaneously encoded even in the absence of a task, and it becomes better factorized in the Mature state. In light of this, an interesting prediction put forward by our model is that also in children we should observe a developmental refinement of numerosity representations (e.g., by RSA), even when there is no task requiring to focus on number. Along this line, a promising venue for future research would be to apply RSA to compare deep networks with human neuroimaging data (for a recent fMRI study on adults, see^8^).

The finding that supervised deep networks trained in visual discrimination tasks may show idiosyncratic (non-human) behavior^61^ has raised concerns about their capacity to faithfully mimic human vision. Here we observed an impressive match between human performance and deep neural networks, which suggests that neurocomputational models based on unsupervised deep learning represent a powerful framework to investigate the emergence of perceptual and cognitive abilities in learning machines that emulate the processing mechanisms of real brains (also see^36^). Furthermore, our exploration of different architectures and learning hyperparameters suggests that numerosity comparison is a challenging task for deep learning models, although the present results do not exclude the possibility that more advanced architectures (e.g., incorporating *ad-hoc* pre-processing stages or convolutional mechanisms) could achieve higher performance. In this respect, it should be noted that even state-of-the-art models, such as those based on generative adversarial networks, have shown unable to explicitly represent numerosity as a fully disentangled factor^62^. The response variability exhibited by the different deep learning architectures also suggests that this framework could be used to study the factors contributing to the emergence of individual differences in human observers, which is crucial for developing personalized computational models that may predict learning outcomes (see^63^ for a recent application to learning to read and dyslexia).

Besides improving our current understanding of the computational foundations of numerosity perception, our modeling work has also the potential for technological applications. For example, intelligent machines that can perceive and manipulate numerosity in a meaningful way would allow to replace human annotators in tedious tasks, such as estimating the number of cells in microscopic images, monitoring crowds and traffic congestion in automatic surveillance systems or the number of trees in aerial images of forests. Number-related questions are also being included in standard benchmarks for assessing intelligent dialogue systems^64^, but the ability to flexibly manipulate numbers and perform quantitative reasoning is still out of reach even for state-of-the-art systems^65^. Indeed, although computers largely outperform humans on tasks requiring the mere application of syntactic manipulations (e.g., performing algebraic operations on large numbers, or iteratively computing the value of a function), they completely lack a conceptual semantics of number.

The problem of grounding symbolic knowledge into some form of intrinsic meaning is well known in artificial intelligence research^66^, and mathematics constitutes one of the most challenging domains for investigating how abstract symbolic notations could be linked to bottom-up, sensorimotor percepts^67^. We believe that our modelling work constitutes a key step towards a better understanding of our visual number sense: a great challenge for future research would be to extend this framework to the realm of formal mathematics^68^, in order to characterize the computational mechanisms underlying the acquisition of symbolic numbers, and their impairments in atypical populations.

## Methods

### Human participants

Forty volunteer students (mean age 23.7 years, range 20–28, 32 females) were recruited at the University of Padova. All participants gave written informed consent to the protocol approved by the Psychological Science Ethics Committee of the University of Padova and did not receive any payment. All experiments were performed in accordance with relevant guidelines and regulations.

### Visual stimuli

Images of size 200 × 200 pixels were generated by randomly placing white dots on a black background. For the discrimination task there were 13 levels of Numerosity (range 7–28), 13 levels of Size (range 2.6–10.4 pixels × 10^5^) and 13 levels of Spacing (range 80–320 pixels × 10^5^), evenly spaced on a logarithmic scale. Note that the range of variability was the same across each orthogonal dimension. For each selected point in the stimulus space 10 different images were generated by randomly varying dots displacement, resulting in a dataset of 21970 unique images. For the human experiment we randomly selected images from the dataset to create 300 image pairs with different magnitude ratios, oversampling the more difficult numerosity ratios (10% with ratio between 0.5 and 0.6; 20% with ratio between 0.6 and 0.7; 30% with ratio between 0.7 and 0.8; 40% with ratio between 0.8 and 0.9). For simulations, 15200 image pairs were created by randomly choosing among all patterns in the dataset. We also created an independent dataset of 65912 images, containing all numbers between 5 and 32, which was used only for unsupervised learning.

### Procedure for the human study

Stimuli were projected on a 19-inch color screen. Participants sat approximately 70 cm from the screen and placed their head on a chin rest. Participants were verbally instructed to select the stimulus with more dots, responding with the left and right arrows of the keyboard depending on its side of appearance (feedback was given only during few practice trials). The task consisted in 3 blocks of 100 trials each, for a total of 300 trials. Each trial began with a fixation cross at the center of the screen (500 ms), followed by the simultaneous presentation of two stimuli (250 ms), one at the right and one at the left of the cross with eccentricity of ~12 visual degrees, and then by two masks of black and white Gaussian noise in the same positions (150 ms). A black screen was then displayed until response, without time limit. After response, a pseudorandom inter-trial interval between 1250 and 1750 ms occurred. Participants also performed a sequential version of the same task, whose outcome was aligned with the simultaneous version and thus it is not further considered (see SI).

### GLM analysis

All responses below stimulus presentation time were considered outliers, as well as response times over two standard deviations from the participant’s mean response time in equally difficult trials (based on numerosity ratio). A generalized linear model (with *probit* link function) was then fitted to the choice data of each participant^41^, which was modeled as a function of the three regressors Numerosity, Size and Spacing:

$$\begin{array}{c}p(ChooseRight)=\\ (1-{\rm{\gamma }})\left(\frac{1}{2}\left[1+erf\left(\frac{{{\rm{\beta }}}_{Side}+{{\rm{\beta }}}_{Num}{\log }_{2}({r}_{num})+{{\rm{\beta }}}_{Size}{\log }_{2}({r}_{size})+{{\rm{\beta }}}_{Spacing}{\log }_{2}({r}_{Spacing})}{\sqrt{2}}\right)\right]-\frac{1}{2}\right)+\frac{1}{2}\end{array}$$

where *r*_*num*_, *r*_*size*_ and *r*_*spacing*_ represent the Numerosity, Size and Spacing ratios between the two stimuli, and the corresponding *β* coefficients represent the degree to which each orthogonal dimension affects discrimination performance. The *β*_*Side*_ coefficient accounts for spatial response biases independent from stimuli properties, while the term *γ* represents the guessing factor accounting for occasional random responses due to distraction. The individual guessing factor was estimated to minimize the deviance of the model and was set to 0.01.

### Projection analysis

The direction of the discrimination vector defined by the coordinates *β*_*Num*,_ *β*_*Size*_ and *β*_*Spacing*_ represents what stimulus features are being mostly used to perform the discrimination, while the magnitude of the vector represents the participant’s acuity in discriminating each feature. In the case of a strategy based exclusively on numerosity the discrimination vector will coincide with the *Numerosity* dimension, and the magnitude of the vector will be exactly *β*_*Num*_. When choice is modulated by other dimensions, the contribution of each individual non-numerical feature is geometrically characterized by projecting the discrimination vector onto each dimension and measuring which one is the closest to the *Numerosity* axis. Similarly, the most representative features can be quantified by measuring the angle between the discrimination vector and each candidate dimension^69^. Multiple comparison tests were always corrected using the Bonferroni method. When the assumption of normality was violated, non-parametric tests (Wilcoxon signed rank) were performed.

### Deep learning

Deep belief networks were first trained in a completely unsupervised way on the stimulus set containing all numbers from 5 to 32 (images were downscaled to 100×100 pixels for computational convenience). Deep networks were built as a stack of two Restricted Boltzmann Machines (RBMs) trained using contrastive divergence^32,70^. Each RBM consisted of two layers of stochastic neurons, fully connected with symmetric weights and without self-connections (SI for details). Visual stimuli were provided as input by clamping the vectorized images on the visible neurons of the first RBM; the subsequent activation of hidden neurons constituted the model’s internal representation of the stimulus. A decision layer was then trained by feeding the internal representations (i.e., the hidden activations of the top RBM) of two paired stimuli to a linear network with two output units, which implemented a binary classification task (note that supervised training did not alter the internal representations of the deep network). We tested 12 different architectures (obtained by varying the number of hidden units in each layer) with 12 different random initializations of the connection weights, for a total of 144 networks. The source code of our simulations is available for download on the Open Science Framework, along with a copy of the trained networks that can be used to further test our model over different types of stimuli and experimental settings^3^^3^.

### Representational similarity analysis

Results reported in the main text refer to the architecture achieving the best numerosity discrimination performance, which had 1500 neurons in the first hidden layer and 1000 neurons in the second layer (control simulations showed that the overall findings hold also if we consider the complete set of architectures). The deep network was probed on a subset of test stimuli created by randomly selecting 10 instances of each combination of three Numerosity levels (7, 18, 28), three Size levels (2.60 × 10^5^, 6.55 × 10^5^, 10.40 × 10^5^) and 3 Spacing levels (0.80 × 10^7^, 2.02 × 10^7^, 3.20 × 10^7^), all equally spaced between the minimum and maximum values in the original stimulus set, for a total of 270 images. The activation patterns of the deepest hidden layer corresponding to instances with the same combination of features were averaged, resulting in 27 mean activation patterns. These mean activation patterns were then compared in order to build a Representational Dissimilarity Matrix (RDM), whose cells contained a number reflecting the dissimilarity (1–Pearson correlation) between the internal representations associated to each combination of stimulus features. For comparison, categorical RDMs corresponding to all possible individual features were built by using as dissimilarity measure the difference between feature values on a log scale. The model RDMs were quantitatively compared to the categorical RDMs using Kendall’s Tau alpha correlation, and their specific relatedness was statistically assessed using one-sided Wilcoxon signed rank tests, considering the simulated RDMs as reference and treating the categorical models as possible candidates. RSA was performed using a publicly available MATLAB toolbox^71^.

## Supplementary information

Supplementary Information.

## Data Availability

All data needed to evaluate the conclusions are present in the paper. The complete source code of the model is available for download at the Open Science Framework: https://osf.io/j7dvc/.

## Footnotes

1 ^1^Although these stimuli do not incorporate the complexity of natural images, which would more faithfully reflect the raw stimuli over which our visual system develops, we argue that such a simplified input could be considered as a fair approximation of the kind of visual representations propagated through the dorsal visual stream, where most of the fine-grained features of the image are discarded but spatial properties of the objects are retained^72^.

2 ^2^Note that we are primarily interested in comparing the *relative change* over development; these two stages might not necessarily correspond to specific points in human development.

3 ^3^https://osf.io/j7dvc
