# Supplementary material for: Visual sense of number vs. sense of magnitude in humans and machines
Source: Sci Rep. 2020 Jun 22;10:10045. doi: 10.1038/s41598-020-66838-5 (PMC7308388; doi:10.1038/s41598-020-66838-5)
Supplement: Supplementary file 1 — Supplementary Information. [file 41598_2020_66838_MOESM1_ESM.pdf]

Supplementary Information for

Visual sense of number *vs.* sense of magnitude in humans and machines

Alberto Testolin[1,2 *], Serena Dolfi[1], Mathijs Rochus[3], Marco Zorzi[1, 4 *]

[1] Department of General Psychology and Padova Neuroscience Center, University of Padova, 35131 Padova, Italy.

[2] Department of Information Engineering, University of Padova, 35131 Padova, Italy.

[3] Department of Experimental Psychology, Ghent University, 9000 Ghent, Belgium.

[4] IRCCS San Camillo Hospital, 30126 Venice-Lido, Italy.

* Correspondence: alberto.testolin@unipd.it, marco.zorzi@unipd.it

This PDF file includes:

Supplementary Methods
Figures S1 to S3
Table S1 and S2
References for SI reference citations

Supplementary Methods

Stimulus space definition

The relationship between the three orthogonal dimensions *Numerosity*, *Size* and *Spacing* and the individual non-numerical features can be algebraically defined according to the following equations, where *n* is the number of items, *TSA* is the total surface area (i.e., the cumulative area of all items), *ISA* is the individual item surface area, *FA* is field area (approximation of convex hull) and *Spar* is sparsity (inverse of density). Note that, after log scaling the axes, the distance between stimulus points in the space is proportional to the ratios of their features, and the equations relating the orthogonal dimensions to the other non-numerical features are all linear equations (DeWind et al. 2015).

$$log_2(n) = log_2\left(\frac{TSA}{ISA}\right) = log_2\left(\frac{FA}{Spar}\right)$$

$$log_2(Size) = log_2(TSA) + log_2(ISA)$$

$$log_2(Spacing) = log_2(FA) + log_2(Spar)$$

Individual features in terms of the three orthogonal dimensions:

$$TSA = \sqrt{Sz \cdot n}$$

$$ISA = \sqrt{\frac{Sz}{n}}$$

$$FA = \sqrt{Sp \cdot n}$$

$$Spar = \sqrt{\frac{Sp}{n}}$$

$$TP = 2\sqrt{\pi} \cdot Sz^{\frac{1}{4}} \cdot n^{\frac{3}{4}}$$

$$IP = 2\sqrt{\pi} \cdot Sz^{\frac{1}{4}} \cdot n^{-\frac{1}{4}}$$

$$Cov = \sqrt{\frac{Sz}{Sp}}$$

$$AC = \sqrt{Sz \cdot Sp}$$

Log of each feature in terms of log of the three orthogonal dimensions:

$$log_2(TSA) = \frac{1}{2}log_2(Sz) + \frac{1}{2}log_2(n)$$

$$log_2(ISA) = \frac{1}{2}log_2(Sz) - \frac{1}{2}log_2(n)$$

$$log_2(FA) = \frac{1}{2}log_2(Sp) + \frac{1}{2}log_2(n)$$
$$log_2(Spar) = \frac{1}{2}log_2(Sp) - \frac{1}{2}log_2(n)$$
$$log_2(TP) = log_2(2\sqrt{\pi}) + \frac{1}{4}log_2(Sz) + \frac{3}{4}log_2(n)$$
$$log_2(IP) = log_2(2\sqrt{\pi}) + \frac{1}{4}log_2(Sz) - \frac{1}{4}log_2(n)$$
$$log_2(Cov) = \frac{1}{2}log_2(Sz) - \frac{1}{2}log_2(Sp)$$
$$log_2(AC) = \frac{1}{2}log_2(Sz) + \frac{1}{2}log_2(Sp)$$

Simulations details

Deep belief networks were implemented as a stack of Restricted Boltzmann Machines (RBMs) (Ackley et al. 1985; Hinton et al. 2006). The dynamics of each RBM was driven by an energy function *E* that specifies which configurations of the neurons are more likely to occur by assigning them a probability value:

$$p(v,h) = \frac{e^{-E(v,h)}}{Z}$$

where *v* and *h* represent the visible and hidden neurons and *Z* is the partition function. Since there are no connections within the same layer the energy function can be defined as:

$$E(v,h) = -b^T v - c^T h - h^T Wv$$

where *W* is the matrix of connections weights and *b* and *c* are the biases of visible and hidden neurons, respectively. RBMs were trained using 1-step contrastive divergence (Hinton 2002). For each training pattern, during the positive phase all visible neurons are clamped to the current pattern, and the activation of hidden neurons is computed by sampling from their conditional probability:

$$P(h|v) = \prod_{j=1}^{n} P(h_j | v)$$

where *n* is the number of neurons in the hidden layer. Conditional activation probabilities for each single neuron are computed using the sigmoid logistic function:

$$P(h_j = 1 | v) = \frac{1}{1 + e^{-b_j - \sum_{i=1}^{m} w_{ij} v_i}}$$

where *m* is the number of neurons in the visible layer. During the negative phase, the activation of the hidden neurons corresponding to the clamped data pattern is similarly used to perform top-down reconstruction of the stimulus over the visible neurons. Connection weights were randomly initialized according to a Gaussian distribution with zero mean and standard deviation of 0.01. Learning hyperparameters were optimized by systematically varying the learning rate (best value 0.15), weight decay factor (best value 0.0001), momentum coefficient (best value 0.7) and mini-batch size (best value 100). Learning was performed using in-house source code optimized for graphic processing units (GPUs) (Testolin et al. 2013).

The model was build using the two-layer architecture adopted in previous studies (Stoianov and Zorzi 2012; Zorzi and Testolin 2018) and explored possible variations by systematically changing the size of the first (H1) and second (H2) hidden layers, resulting in 12 combinations:

	N.1	N.2	N.3	N.4	N.5	N.6	N.7	N.8	N.9	N.10	N.11	N.12
**H1**	500	500	500	500	1000	1000	1000	1500	1500	1500	1500	1500
**H2**	500	1000	1500	2000	500	1000	1500	2000	500	1000	1500	2000

The supervised read-out layer was trained using an efficient implementation of linear associative learning formalized according to the pseudoinverse method (Hertz et al. 1991; Zorzi et al. 2013). The read-out layer simultaneously received the pattern of activity elicited by two different input images and was trained to assess which of the two contained the larger numerosity, as in (Cappelletti et al. 2014; Zorzi and Testolin 2018). 15200 training pairs were used to train the read-out, while a different combination of 15200 image pairs was used to test it. It should be noted that the model's test set thus more systematically covered the stimulus space, compared to the human's test set that only included 300 image pairs. It should also be noted that the classifier weights were not further tuned during testing, thus our model does not take into account practice effects that might occur during human testing.

Simulation of hysteresis effect

In order to investigate whether the difficulty of preceding trials could produce an effect in the final numerosity discrimination performance, for the 12 instances of the best performing deep network architecture we implemented a proof-of-concept simulation where classifier training was made iterative (i.e., the weights were adjusted after each single trial) and the sequence of training patterns was manipulated according to three conditions:

1) in the "Easy to Hard" condition, the classifier first learned to discriminate easy ratios and then gradually progressed toward more difficult ratios;
2) in the "Hard to Easy" condition, the order was reversed so that harder trials were shown at the beginning of training;
3) in the baseline "Random" condition, all trials were shuffled and presented randomly.

To create such progression, we generated five different subsets of 1000 image pairs, containing increasingly more difficult ratios: 0.50, 0.66, 0.75, 0.80, 0.88. The iterative version of the classifier was implemented using the Delta rule, and each subset was learned for 100 epochs before moving to the next one. After training, the classifier was tested on a left-out test set containing all ratios. As shown in Fig. S2 and Table

S2, the Mature network achieves a better final performance in the "Easy to Hard" condition compared to the "Random" baseline [$t(22) = 3.37$, $p < .01$], while the "Hard to Easy" condition leads to a worsening in acuity compared to the baseline [$t(22) = 3.09$, $p < .01$].

Representational Similarity Analysis (RSA)

RSA was carried out using the methods and toolbox described in (Kriegeskorte 2008; Nili et al. 2014). The activation patterns of the deepest hidden layer in response to the selected stimuli were extracted and averaged across instances of the same combination of features, resulting in 27 mean activation patterns. These mean activation patterns were then compared, computing a Representational Dissimilarity Matrix (RDM)[1]. The latter is a symmetric matrix containing pairwise dissimilarity measures between the internal representations associated to each combination of stimulus features. Given the number of stimuli, our resulting RDMs were 27x27 matrices encoding dissimilarity as 1 – Pearson correlation between each pair of representations. For comparison, categorical models for every individual stimulus feature were built by computing artificial RDMs encoding dissimilarity between the stimuli as their pairwise difference in that feature on a logarithmic scale. Categorical models were created for numerosity, field area, total surface area, item surface area, sparsity, convex hull, total perimeter and item perimeter (Fig. 4A in the main text).

The categorical RDMs were quantitatively compared to the model RDMs using Kendall's rank correlation, and their specific relatedness was statistically assessed, separately for Young and Mature models, by computing the correlation between each categorical RDM and the separate instances of the model RDMs and performing a one-sided Wilcoxon signed rank test against the hypothesis of no correlation (Fig. 4C). We also performed the same analysis using alternative tests available in the RSA toolbox (i.e., randomization and bootstrapping), obtaining similar results. Multiple comparisons were corrected using False Discovery Rate, setting to 0.01 the expected proportion of categorical RDMs falsely declared significant among all candidate RDMs declared significant. Pairwise comparisons between categorical RDMs were also conducted to assess possible differences in their relatedness to the model RDMs, separately for Young and Mature networks: for each pair of categorical RDMs, two-sided Wilcoxon signed rank tests were performed comparing their correlation with the model RDMs, against the null hypothesis of an equal correlation with the model (Fig. S3). Correction for multiple comparisons was carried out using FDR with threshold 0.01. RDMs shown in Fig. 4A are colored according to the standard toolbox colormap "blue-cyan-gray-red-yellow".

t-Distributed Stochastic Neighbor Embedding (t-SNE)

t-SNE projects high-dimensional objects in a two-dimensional space, with the goal of mapping with high probability similar objects into nearby points and dissimilar objects into distant points. The algorithm comprises two main stages. First, it constructs a probability distribution over pairs of high-dimensional objects in such a way that similar objects have a high probability of being picked, whilst dissimilar points

[1] An alternative method would be to compute the dissimilarity between every pair of activation vectors, and then average the values corresponding to the same combination of features. However, we verified that the resulting RDMs were not significantly different (average Pearson correlation between RDMs calculated using the two different approaches was greater than 0.9, both for Young and Mature models).

have a very low probability of being picked. Second, it defines a similar probability distribution over the points in the low-dimensional map, and it minimizes the Kullback–Leibler divergence between the two distributions with respect to the locations of the points in the map (Maaten and Hinton 2008).

In our analysis, we considered the best performing deep learning architecture and we used as high-dimensional objects the activation of the neurons in the second hidden layer corresponding to each stimulus, divided according to four different congruency conditions: 1) Numerosity, Size and Spacing all congruent; 2) Numerosity and Size congruent, Spacing incongruent; 3) Numerosity and Spacing congruent, Size incongruent; 4) Size and Spacing congruent, Numerosity incongruent. As in common practice, the t-SNE cluster analysis was performed on the first 20 components extracted from a principal component analysis over the activation's matrix. Distances between data points were measured using Euclidian distance and the perplexity parameter was set to 100. In the resulting two-dimensional plot (see Fig. 5 in the main text) the display numerosity is represented using different colors, according to a standard JET colormap.

Simultaneous *vs.* sequential presentation of the stimuli

All subjects involved in the study also carried out a sequential version of the numerosity discrimination task. A fixation cross was presented for 500 ms; then a first cloud of dots appeared on the left or on the right of fixation for 250 ms, followed by a mask lasting for 150 ms and then by a second cloud of dots on the opposite side of the fixation, again for 250 ms. As for the simultaneous task, a final mask of 150 ms was then presented. The side of appearance of the first array was randomized. A black response screen was then presented until subject's response. The inter-trial interval ranged between 1250 and 1750 ms.

Discrimination accuracy was well above chance (86.13%, range: 65.85-93%). Comparing the performance in the two versions of the numerosity comparison task, a two-by-two repeated measures ANOVA revealed a significant difference in accuracy between the simultaneous and sequential tasks ($F(1,36) = 9.23$, $p < .01$, $\eta^2 = 0.2$) but no significant effect of task order and no interaction between order presentation and task variant. The GLM fit for the sequential task was significant at individual subject level (mean adjusted $R^2 = .60$, mean chi-square value = 211.17, all $p < .001$). Coefficient fits for each orthogonal feature were significantly different from zero for *βNum* ($t(37) = 21.45$, $p < .001$), *βSize* ($t(37) = -2.71$, $p < .05$) and *βSpacing* ($t(37) = 3.67$, $p < .001$). The projection analysis comparing *βNum* to coefficient weights of all the other features revealed that *βNum* was significantly larger than all other projections (all ts > 9.53, all ps < .001). Coherently with the projection results, in the angle analysis Numerosity resulted the closest dimension to the discrimination vector (8.71 deg), followed by Total Perimeter (22.21 deg), with a significant angle between these two closest features ($z = 5.23$, $p < .001$). Comparing the two task variants, the angle between the discrimination vector and the Numerosity dimension resulted statistically larger in the simultaneous task than in the sequential task ($t = 2.14$, $p < .05$, $d = 0.35$), whose discrimination vector resulted instead significantly farther from the closest non-numerical feature (Total perimeter) than in the simultaneous task ($t = 4.09$, $p < .001$, $d = 0.66$).

Note that our simulations focused on the data collected with the simultaneous task, because the latter is more consistent with the way the comparison task is implemented in the model. Indeed, to carry out the numerosity comparison the decision layer receives as input the internal representation of two stimuli at the same time.

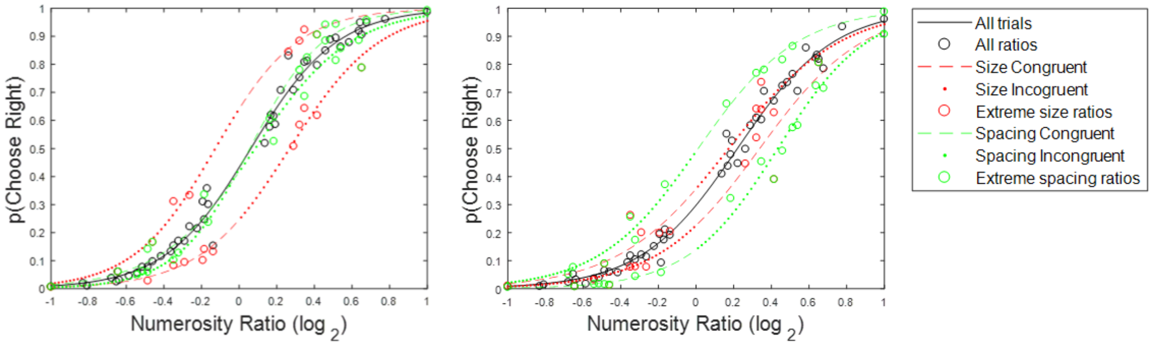

Figure S1. Model fit for two participants. The probability of choosing the right stimulus array is modeled as a function of the log of Numerosity, Size and Spacing ratios. Black lines indicate the model fit for all data (black circles). Red color shows data and model fits for the trials with extreme Size ratio, while green color shows data and model fit for trials with great Spacing ratio. The influence of Size and Spacing are determined by the offset between the colored lines and the original model fit, with a positive or negative effect on performance depending on the congruency between non-numerical features and number: dashed lines indicate that Size or Spacing were congruent with numerosity, while dotted lines indicate incongruent trials.

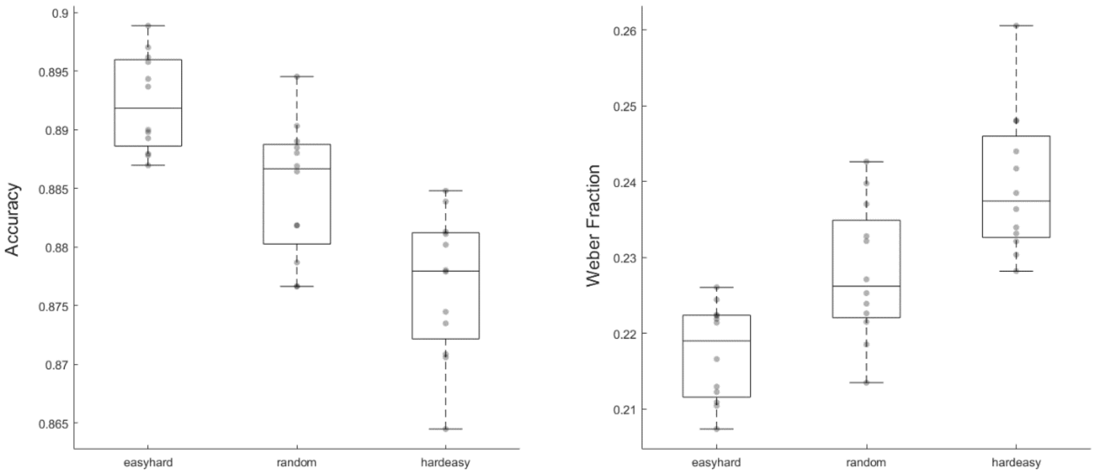

Figure S2. Demonstration of hysteresis effect for the 12 instances of the best performing Mature network. When training progresses from easy to hard ratios, the model achieves a significantly higher accuracy (left panel) and a smaller Weber fraction (right panel) compared to the baseline "Random" condition. When training progresses from hard to easy ratios, final performance instead significantly decreases compared to the baseline.

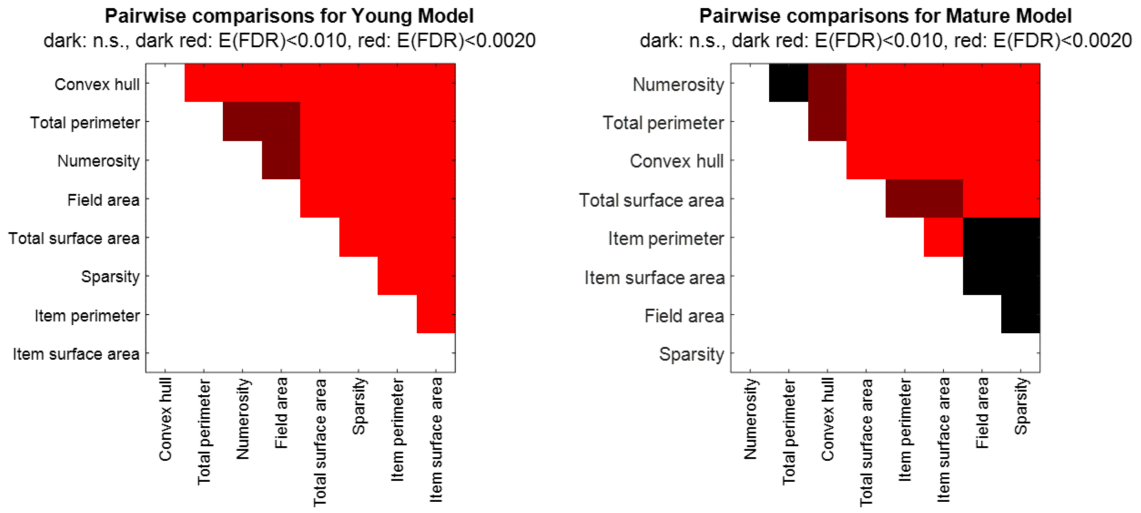

Figure S3. Pairwise comparisons between all candidate models in their relatedness to the reference RDM. The matrix shows the results of two-sided Wilcoxon signed rank tests for each pair of categorical RDM. Significance is based on different FDR thresholds, encoded using different levels of red: black indicates nonsignificant comparisons. CH: Convex Hull; TP: Total Perimeter; FA: Field Area; TSA: Total Surface Area; Spar: Sparsity; IP: Item Perimeter; ISA: Item Surface Area.

	Task Num				*Task Size*				*Task Spacing*			
	Acc	β_{Num}	β_{Size}	$\beta_{Spacing}$	*Acc*	β_{Num}	β_{Size}	$\beta_{Spacing}$	*Acc*	β_{Num}	β_{Size}	$\beta_{Spacing}$
**Young**	0.83 (0.01)	2.55 (0.06)	0.60 (0.03)	0.33 (0.04)	0.56 (0.01)	0.75 (0.05)	0.24 (0.02)	-0.08 (0.02)	0.76 (0.01)	0.40 (0.03)	-0.05 (0.02)	1.17 (0.04)
**Mature**	0.91 (0.01)	3.74 (0.09)	0.09 (0.04)	0.14 (0.02)	0.83 (0.01)	0.07 (0.01)	1.84 (0.05)	-0.01 (0.02)	0.74 (0.01)	0.16 (0.03)	0.04 (0.02)	1.02 (0.04)

Table S1: Read-out accuracy (Acc) and beta coefficients for Young and Mature network, considering three different classification tasks: Numerosity discrimination, Size discrimination, and Spacing discrimination. Results are averaged across all 12 instances of the architecture selected for the RSA (standard deviation in brackets).

	Easy to Hard		*Random*		*Hard to Easy*	
	Acc	*Wf*	*Acc*	*Wf*	*Acc*	*Wf*
**Young**	0.837 (0.005)	0.306 (0.009)	0.836 (0.005)	0.306 (0.010)	0.837 (0.004)	0.305 (0.007)
**Mature**	0.892 (0.004)	0.217 (0.006)	0.885 (0.006)	0.228 (0.009)	0.877 (0.006)	0.240 (0.009)

Table S2: Read-out accuracy (*Acc*) and Weber fraction (*Wf*) showing the hysteresis effect for the Mature network (standard deviation in brackets).

Supplementary References

Ackley D, Hinton GE, Sejnowski TJ. 1985. A learning algorithm for Boltzmann machines. Cogn Sci. 9:147–169.

Cappelletti M, Didino D, Stoianov I, Zorzi M. 2014. Number skills are maintained in healthy ageing. Cogn Psychol. 69:25–45.

DeWind NK, Adams GK, Platt ML, Brannon EM. 2015. Modeling the approximate number system to quantify the contribution of visual stimulus features. Cognition. 142:247–265.

Hertz JA, Krogh AS, Palmer RG. 1991. Introduction to the theory of neural computation. Redwood City, CA: Addison-Weasley.

Hinton GE. 2002. Training products of experts by minimizing contrastive divergence. Neural Comput. 14:1771–1800.

Hinton GE, Osindero S, Teh Y. 2006. A fast learning algorithm for deep belief nets. Neural Comput. 18:1527–1554.

Kriegeskorte N. 2008. Representational similarity analysis – connecting the branches of systems neuroscience. Front Syst Neurosci. 2:1–28.

Maaten L Van Der, Hinton GE. 2008. Visualizing data using t-SNE. J Mach Learn Res. 9:2579–2605.

Nili H, Wingfield C, Walther A, Su L, Marslen-Wilson W, Kriegeskorte N. 2014. A Toolbox for Representational Similarity Analysis. PLoS Comput Biol. 10.

Stoianov I, Zorzi M. 2012. Emergence of a “visual number sense” in hierarchical generative models. Nat Neurosci. 15:194–196.

Testolin A, Stoianov I, De Filippo De Grazia M, Zorzi M. 2013. Deep unsupervised learning on a desktop PC : A primer for cognitive scientists. Front Psychol. 4:251.

Zorzi M, Testolin A. 2018. An emergentist perspective on the origin of number sense. Philos Trans R Soc B Biol Sci. 373.

Zorzi M, Testolin A, Stoianov IPIP. 2013. Modeling language and cognition with deep unsupervised learning: a tutorial overview. Front Psychol. 4:515.
